# Prediction tool for disability progression and mortality in older adults eligible for Japanese long-term care insurance: Koriyama study

**DOI:** 10.1097/MD.0000000000033103

**Published:** 2023-03-03

**Authors:** Takaaki Fujita, Kazuaki Iokawa

**Affiliations:** a Department of Occupational Therapy, School of Health Sciences, Fukushima Medical University, Fukushima, Japan.

**Keywords:** insurance, Japan, long-term care, mortality, prediction model

## Abstract

This study aims to create a simple model for predicting disability progression and death among older adults with Japanese long-term care insurance certification. This retrospective study analyzed the anonymized data provided by Koriyama City. The participants were 7706 older adults who were initially certified to be support levels 1 and 2 or care levels 1 and 2 for the purpose of obtaining Japanese long-term care insurance. The results of the certification questionnaire at the initial survey stage were used to create decision tree models intended to predict whether disability progression and death would occur within 1 year. In support levels 1 and 2, among those who scored both “daily decision making” item as other than “possible” and the “taking drugs” item as other than “independent,” 64.7% had an adverse outcome. In care levels 1 and 2, among those who scored both the “shopping” item as “totally dependent” and the “defecation” item as other than “independent,” 58.6% had an adverse outcome. The accuracy of classification of the decision trees were 61.1% in support levels 1and 2, and 61.7% in care levels 1 and 2. The overall accuracy of the decision tree is low, making it impractical to use it for all subjects. Nevertheless, based on the results of the 2 assessments in this study, the process of identifying a particular group of older adults at a high risk of an increased need for long-term care or possible death within a year is very simple and useful.

## 1. Introduction

Population aging is a global phenomenon, and virtually every country in the world is experiencing both relative and absolute growth in the older population.^[1]^ Of all the countries experiencing population aging, Japan is the country with the highest percentage of elderly persons, with that share reaching 28.8% in 2020.^[2]^ The aging of Japan’s population will continue to accelerate, with 1 in 2.6 people predicted to be 65 years old and above, and 1 in 3.9 to be 75 years old and above, in 2065.^[2]^ In Japan, the long-term care insurance system was launched in 2000 as a social safety net to support the nursing care needs of the elderly. Under this system, people aged 65 and above can receive nursing care services according to their level of needed care (7 level: support level 1–2, care level 1–5) regardless of the cause.^[3]^ Similar long-term care insurance systems are in place in Germany and South Korea.^[4,5]^

In Japan, reform of the long-term care insurance system in 2005 led to a shift from a system that emphasized “nursing care” to a system that emphasized “prevention” to prevent aggravation of the need for long-term care. Nevertheless, the number of people certified for long-term care insurance in Japan has increased 35% (from about 5.06 million to 6.82 million) between 2011 and 2021.^[6]^ In addition, the cost of nursing care services has increased by 40%.^[6]^ Those whose care needs are mild tend to be more likely to experience disability progression, and those whose need for nursing care is more severe are at higher risk of death and pay more for nursing care services.^[6,7]^ Therefore, in Japan, it is particularly important to avoid aggravating the level of nursing care needs of people with mild disabilities.

To prevent aggravation of the degree of nursing care and improve the survival rate of those older adults who have received long-term care insurance certification, it is important to clarify the risk factors and to identify those who require early intervention. Studies on Japanese participants reported that grip strength,^[8]^ memory impairment,^[9,10]^ activities of daily living (ADLs; mobility, nail trimming, bathing, urinary, etc),^[10]^ and use of home care services^[11,12]^ can predict aggravation of the level of nursing care. In addition, aggravation of the degree of nursing care leads to an increased risk of death.^[13]^ However, despite gradual clarification of the risk factors for the aggravation of the level of the need for long-term care and death among older adults, few prediction tools have been created to identify those who need early intervention.

Therefore, this study is intended to create a prediction tool and death among older adults who have received long-term care insurance certification, especially those with mild conditions. Because people with diverse backgrounds are involved in supporting the older adults who have received long-term care insurance certification, the prediction tool should be simple and practical enough for anyone to use. Therefore, we tried to create a simple model in this study.

## 2. Materials and methods

**Figure 1.**
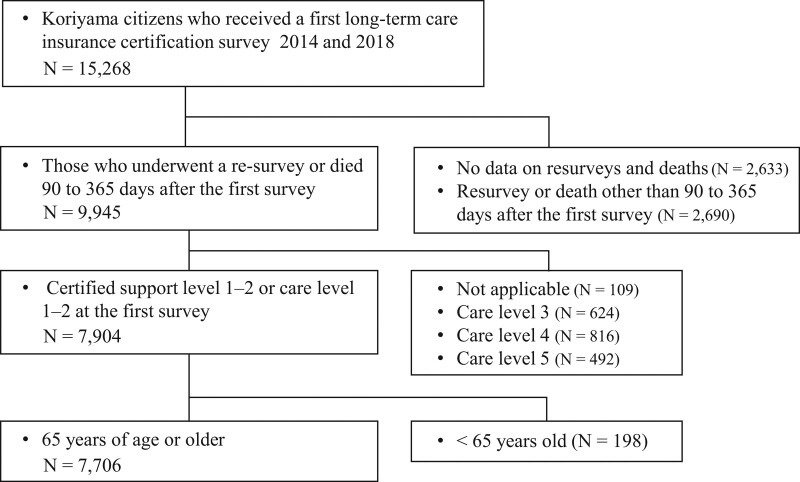
Flow diagram for study participants.

The present study was a joint research project between Koriyama City and Fukushima Medical University to promote the Sustainable Development Goals Experience Future City Koriyama All-Generation Healthy Urban Area Creation Project. This study was a retrospective study, which analyzed the anonymized data provided by Koriyama City. Therefore, there was no need to obtain informed consent or disclose information. This study was approved by the Fukushima Medical University Ethics Committee (general 2021-114). Of the 15,268 residents of Koriyama who first applied for long-term care certification between fiscal 2014 and 2018, 7706 met the inclusion criteria. Included were; Those who underwent a resurvey of long-term care certification or died between 90 and 365 days after the first survey; Those who were assessed at support level 1 or 2 or care levels 1 or 2 at the time of the first application, and; Those who were 65 years old or older (Fig. 1). In the first accreditation case, the valid period of accreditation is 6 months in principle; however, if the municipality deems it necessary, it is stipulated to be between 3 and 12 months. Therefore, those who underwent a resurvey of long-term care certification other than 90 to 365 days after receiving their first long-term care certification were excluded from this study, as they were considered to have special circumstances. Similarly, those who died outside the 90 to 365-day period were also excluded from the survey. However, no participants were excluded from the study because of missing data.

When certifying the need for long-term care, a primary judgment is first made based on the family doctor’s report and the results of the certification survey that verifies the applicant’s mental and physical condition by a representative from the municipality. The subsequent required level of care is decided by the long-term care certification committee consisting of academic health, medical, and welfare experts, based on the results of the primary judgment, particular matters, and the doctor’s report. Support level 1 and support level 2, care level 1, and care level 2 refer to the conditions under which care is required for 25 to 32 minutes, 32 to 50 minutes, and 50 to 70 minutes per day, respectively.

In this study, we defined the aggravation of the need for long-term care or death within 1 year (between 90 and 365 days after the initial certification survey) as an adverse event and attempted to create a model to predict it. We created separate prediction tools for older adults in the support level 1 and 2 group and in the care level 1 and 2 group. Considering the practicality of the model, the variables used in the prediction tools were age, gender, and the results of the basic items of the certification questionnaire that were routinely administered in the certification survey: 13 items of physical function, 12 items for ADLs, 9 items of cognitive function, 15 items for psychological and behavioral disorder, and 6 items of adaptation to social life. The basic items of this certification questionnaire evaluate the degree of independence of various functions on a scale of 2 to 4.^[3]^

First, the subjects were classified into groups with and without adverse events, and age, gender, and basic items of the certified questionnaire were compared between the 2 groups using the t-test, chi-square test, Fisher exact test, and Mann–Whitney test. Next, a stepwise logistic regression was performed to select candidate variables that were associated with the adverse events. The independent variables were items observed significant difference between groups and the dependent variable was the presence or absence of adverse events. Last, decision trees (classification and regression trees) were performed to create a simple and practical prediction tool, which is the purpose of this study. The decision tree is a simple prognostic prediction tool because it does not use mathematical formulas, and the results are presented in chart form and are easy to interpret. Variables selected in the stepwise logistic regression were used in the decision tree. To prevent overfitting, cost complexity pruning (±1 standard deviation) was performed, and the minimum number of cases from the parent and child nodes was set at 2% and 1% of sample size. Tree maximum depth was set at 3. All statistical analyses were performed using version 25 of the IBM SPSS statistics (IBM Corporation, Armonk, NY), and *P* < .05 was considered statistically significant.

## 3. Results

The participants of this study were 82.1 ± 6.9 years old, and 61.8% were female (Table 1). Among older adults, 2795 were in support level 1; 1286 were in support level 2; 2614 were in care level 1; and 1011 were in care level 2 in the first certification survey. Among all participants, the incidence of adverse events within 1 year was 43.6% (disability progression, 32.1%; death, 11.5%). In support levels 1 and 2 and care levels 1 and 2, the incidence of adverse events were 46.9% (disability progression, 38.3%; death 8.6%) and 39.9% (disability progression, 25.1%; death, 14.8%), respectively.

**Table 1:** Participant’s age, gender, and results of first long-term care insurance certification survey.

	Overall	Support level 1 and 2	Care level 1 and 2
Age	82.1 ± 6.9	82.0 ± 6.7	82.2 ± 7.1
Gender, women, %	61.8	65.0	58.3
Duration between first to resurvey	298.3 ± 62.0	299.3 ± 60.4	297.2 ± 63.8
Physical function			
Paralysis, not exist, %	69.6	70.6	68.4
Contracture, not exist, %	89.9	88.5	89.4
Roll over in the bed, possible†, %	93.1	94.2	91.8
Sitting up in the bed, possible†, %	98.6	99.5	97.5
Keep sitting, possible†, %	92.5	94.9	89.8
Hold standing position with two legs, possible†, %	98.9	99.7	98.0
Walking, possible†, %	93.1	98.7	86.9
Standing up, possible†, %	99.4	99.8	98.9
Hold standing position with one legs, possible†, %	94.1	98.3	89.3
Washing oneself, independent, %	59.5	77.4	39.4
Cutting nails, independent, %	60.9	74.5	45.6
Acuity, normal, %	94.0	94.4	93.5
Hearing, normal, %	63.7	64.9	62.5
ADL function			
Transfer, independent, %	94.9	99.7	98.8
Mobility, independent, %	92.3	99.3	84.3
Swallowing, independent, %	89.8	89.7	90.0
Eating, independent, %	98.1	99.5	96.5
Urination, independent, %	87.3	97.8	75.6
Defecation, independent, %	90.4	98.8	81.0
Oral hygiene, independent, %	86.2	98.2	72.8
Washing face, independent, %	81.6	95.2	66.2
Hairdressing, independent, %	92.6	98.7	85.6
Wearing on/off shirt and jacket, independent, %	83.4	97.1	68.0
Wearing on/off pants, trousers, and skirt, independent, %	82.6	97.3	66.1
Going outdoor, more than once a week, %	49.9	61.7	36.6
Cognitive function			
Communication, possible, %	96.5	98.7	94.1
Understanding of routine, possible, %	95.8	99.8	91.3
Say birthday and age, possible, %	97.6	99.7	95.3
Short term memory, possible, %	72.7	91.8	51.3
Say one’s name, possible, %	100.0	100.0	99.9
Understanding of current season, possible, %	90.5	98.4	81.5
Understanding of place, possible, %	98.9	100.0	97.7
Wandering, no, %	99.1	100.0	98.1
Difficulty to return home, no, %	98.6	99.7	97.3
Behavioural and psychological symptom of dementia related problems			
Persecution tendency, not exist, %	94.4	98.7	89.6
Confabulation, not exist, %	92.3	98.1	85.8
Rapid changes in moods, not exist, %	92.0	97.0	86.4
Reversal of the night-day sleep-wake cycle, not exist, %	97.0	99.5	94.3
Repeat the same story, not exist, %	83.6	94.8	71.0
Shouting, not exist, %	96.3	98.8	93.5
Resistance to care, not exist, %	99.4	99.9	98.8
Restless, not exist, %	98.9	99.9	97.7
Eager to go out by oneself, not exist, %	99.4	100.0	98.8
Collection mania, not exist, %	99.2	99.9	98.4
Violence, not exist, %	99.5	100.0	99.0
Severe memory loss, not exist, %	56.1	65.6	45.3
Abnormal muttering and laughing to oneself, not exist, %	97.5	99.5	95.3
Selfish behaviour, not exist, %	99.5	99.9	99.0
Endless talking, not exist, %	98.3	99.6	96.9
Adaptation to social life			
Taking drugs, independent, %	42.0	68.6	12.1
Handling finance, independent, %	53.6	75.6	28.3
Daily decision making, possible, %	27.3	41.3	11.6
Difficulty of adaptation to group activity, not exist, %	100.0	100.0	100.0
Shopping, independent, %	16.9	26.5	6.2
Cooking, independent, %	30.3	42.6	16.5

† possible with or without using upper limb.

In support levels 1 and 2, a comparison between adverse and non-adverse event groups revealed significant differences in age, gender, and 22 items of the certification questionnaire (5 items of physical function, 4 items of ADLs function, 5 items of cognitive function, 3 items of psychological and behavioral disorder, and 5 items of adaptation to social life, Table 2). As a result of stepwise logistic regression analysis using these variables as dependent variables, age, the following items were selected: “roll over in bed,” “acuity,” “urination,” “going out,” “short-term memory,” “severe memory loss,” “taking drugs,” “handling finances,” “daily decision making,” and “shopping” (*P* < .05, Table 3).

**Table 2:** Comparison of functions between non-and adverse outcomes groups.

	Support level 1 and 2			Care level 1 and 2		
	Non adverse outcomes	Adverse outcomes	*P* value	Non adverse outcomes	Adverse outcomes	*P* value
Age	81.6 ± 6.5	82.5 ± 6.8	<.01	81.9 ± 6.9	82.6 ± 7.3	<.01
Gender, women, %	68.1	61.5	<.01	61.8	65.0	<.01
Physical function						
Paralysis, not exist, %	69.4	72.0	.07	68.7	68.0	.66
Contracture, not exist, %	87.7	89.3	.12	89.8	88.7	.26
Roll over in the bed (score range 1–3)	1 (1–2)	1 (1–2)	<.01	1 (1–2)	1 (1–2)	.59
Sitting up in the bed (score range 1–3)	2 (2–2)	2 (2–2)	<.05	2 (2–2)	2 (2–2)	<.01
Keep sitting (score range 1–4)	1 (1–2)	1 (1–2)	.21	1 (1–2)	1 (1–2)	<.01
Hold standing position with two legs (score range 1–3)	1 (1–1)	1 (1–1)	.77	1 (1–2)	1 (1–2)	.38
Walking (score range 1–3)	1 (1–2)	1 (1–2)	.34	2 (1–2)	2 (1–2)	<.05
Standing up (score range 1–3)	2 (2–2)	2 (2–2)	<.05	2 (2–2)	2 (2–2)	<.01
Hold standing position with one leg (score range 1–3)	2 (1–2)	2 (2–2)	.37	2 (1–2)	2 (2–2)	<.01
Washing oneself (score range 1–4)	1 (1–1)	1 (1–1)	.19	2 (1–2)	2 (1–3)	<.01
Cutting nails (score range 1–3)	1 (1–2)	1 (1–2)	.76	2 (1–3)	2 (1–3)	<.01
Acuity (score range 1–5)	1 (1–1)	1 (1–1)	<.01	1 (1–1)	1 (1–1)	.10
Hearing (score range 1–5)	1 (1–2)	1 (1–2)	<.01	1 (1–2)	1 (1–2)	<.01
ADL function						
Transfer (score range 1–4)	1 (1–1)	1 (1–1)	.09	1 (1–1)	1 (1–1)	.32
Mobility (score range 1–4)	1 (1–1)	1 (1–1)	.14	1 (1–1)	1 (1–1)	.20
Swallowing (score range 1–3)	1 (1–1)	1 (1–1)	.41	1 (1–1)	1 (1–1)	<.05
Eating (score range 1–4)	1 (1–1)	1 (1–1)	.17	1 (1–1)	1 (1–1)	<.01
Urination (score range 1–4)	1 (1–1)	1 (1–1)	<.01	1 (1–1)	1 (1–3)	<.01
Defecation (score range 1–4)	1 (1–1)	1 (1–1)	<.05	1 (1–1)	1 (1–1)	<.01
Oral hygiene (score range 1–3)	1 (1–1)	1 (1–1)	<.01	1 (1–2)	1 (1–2)	<.01
Washing face (score range 1–3)	1 (1–1)	1 (1–1)	.77	1 (1–2)	1 (1–2)	.21
Hairdressing (score range 1–3)	1 (1–1)	1 (1–1)	.09	1 (1–1)	1 (1–1)	<.01
Wearing on/off shirt and jacket (score range 1–4)	1 (1–1)	1 (1–1)	.07	1 (1–3)	1 (1–3)	<.01
Wearing on/off pants, trousers, and skirt (score range 1–4)	1 (1–1)	1 (1–1)	.91	1 (1–3)	1 (1–3)	<.01
Going outdoor (score range 1–3)	1 (1–2)	1 (1–2)	<.01	2 (1–3)	2 (1–3)	<.01
Cognitive function						
Communication (score range 1–4)	1 (1–1)	1 (1–1)	<.01	1 (1–1)	1 (1–1)	
Understanding of routine, possible, % (score range 1–2)	100.0	99.6	<.05	93.5	88.1	<.01
Say birthday and age, possible, % (score range 1–2)	100.0	99.3	<.01	96.3	93.9	<.01
Short term memory, possible, % (score range 1–2)	95.8	87.2	<.01	52.0	50.2	.28
Say one’s name, possible, % (score range 1–2)	100.0	100.0	1.00	100.0	99.9	.16
Understanding of current season, possible, % (score range 1–2)	99.4	97.3	<.01	84.7	76.8	<.01
Understanding of place, possible, % (score range 1–2)	100.0	99.9	.47	98.2	96.9	<.01
Wandering (score range 1–3)	1 (1–1)	1 (1–1)	.13	1 (1–1)	1 (1–1)	.62
Difficulty to return home (score range 1–3)	1 (1–1)	1 (1–1)	.11	1 (1–1)	1 (1–1)	.66
Behavioural and psychological symptom of dementia related problems						
Persecution tendency (score range 1–3)	1 (1–1)	1 (1–1)	.25	1 (1–1)	1 (1–1)	.40
Confabulation (score range 1–3)	1 (1–1)	1 (1–1)	<.01	1 (1–1)	1 (1–1)	.44
Rapid changes in moods (score range 1–3)	1 (1–1)	1 (1–1)	.14	1 (1–1)	1 (1–1)	.74
Reversal of the night-day sleep-wake cycle (score range 1–3)	1 (1–1)	1 (1–1)	.35	1 (1–1)	1 (1–1)	<.05
Repeat the same story (score range 1–3)	1 (1–1)	1 (1–1)	<.01	1 (1–3)	1 (1–2)	<.01
Shouting (score range 1–3)	1 (1–1)	1 (1–1)	.12	1 (1–1)	1 (1–1)	.48
Resistance to care (score range 1–3)	1 (1–1)	1 (1–1)	.49	1 (1–1)	1 (1–1)	.07
Restless (score range 1–3)	1 (1–1)	1 (1–1)	.26	1 (1–1)	1 (1–1)	.08
Eager to go out by oneself (score range 1–3)	1 (1–1)	1 (1–1)	1.00	1 (1–1)	1 (1–1)	.75
Collection mania (score range 1–3)	1 (1–1)	1 (1–1)	.49	1 (1–1)	1 (1–1)	.63
Violence (score range 1–3)	1 (1–1)	1 (1–1)	.35	1 (1–1)	1 (1–1)	.70
Severe memory loss (score range 1–3)	1 (1–2)	1 (1–3)	<.01	2 (1–3)	2 (1–3)	<.05
Abnormal muttering and laughing to oneself (score range 1–3)	1 (1–1)	1 (1–1)	.71	1 (1–1)	1 (1–1)	<.05
Selfish behaviour (score range 1–3)	1 (1–1)	1 (1–1)	.26	1 (1–1)	1 (1–1)	.58
Endless talking (score range 1–3)	1 (1–1)	1 (1–1)	.62	1 (1–1)	1 (1–1)	.22
Adaptation to social life						
Taking drugs (score range 1–3)	1 (1–1)	1 (1–2)	<.01	2 (2–2)	2 (2–2)	<.01
Handling finance (score range 1–3)	1 (1–1)	1 (1–2)	<.01	2 (1–3)	2 (2–3)	<.01
Daily decision making (score range 1–4)	2 (1–2)	1 (2–2)	<.01	2 (2–2)	2 (2–2)	<.01
Difficulty of adaptation to group activity (score range 1–3)	1 (1–1)	1 (1–1)	.35	1 (1–1)	1 (1–1)	1.00
Shopping (score range 1–4)	4 (1–4)	4 (3–4)	<.01	4 (4–4)	4 (4–4)	<.01
Cooking (score range 1–4)	3 (1–4)	4 (1–4)	<.01	4 (4–4)	4 (4–4)	<.01

Mean ± standard deviation, or median (25–75%tile), or %.

ADL = activities of daily living.

**Table 3:** Logistic regression analysis with adverse events as the dependent variable.

	Support level 1 and 2		Care level 1 and 2	
	Odds ratio (95% CI)	*P* value	Odds ratio (95% CI)	*P* value
Age	1.01 (1.00–1.02)	<.05	1.02 (1.01–1.03)	<.01
Roll over in the bed	0.80 (0.71–0.89)	<.01		
Acuity	1.21 (1.03–1.43)	<.05		
Urination	1.43 (1.13–1.82)	<.01		
Going outdoor	1.13 (1.01–1.26)	<.05		
Say birthday and age	7.17 (0.91–56.80)	.06		
Short term memory	1.81 (1.38–2.37)	<.01		
Severe memory loss	1.23 (1.13–1.34)	<.01		
Taking drugs	1.36 (1.16–1.59)	<.01		
Handling finance	1.19 (1.04–1.35)	<.01	1.22 (1.12–1.34)	<.01
Daily decision making	1.44 (1.25–1.67)	<.01		
Shopping	1.17 (1.11–1.24)	<.01		
Gender			0.62 (0.54–0.71)	<.01
Standing up			1.98 (1.45–2.72)	<.01
Washing oneself			1.12 (1.05–1.19)	<.01
Swallowing			1.26 (1.00–1.57)	<.05
Eating			1.29 (1.03–1.61)	<.05
Defecation			1.19 (1.09–1.29)	<.01
Understanding of routine			1.34 (1.03–1.75)	<.05
Understanding of current season			1.46 (1.21–1.77)	<.01
Abnormal muttering and laughing to oneself			1.19 (1.01–1.41)	<.05

In care levels 1 and 2, significant differences were seen in age, gender, and 30 items of certification questionnaire between groups (8 items of physical function, 9 items of ADLs function, 4 items of cognitive function, 4 items of psychological and behavioral disorder, and 5 items of adaptation to social life). As a result of stepwise logistic regression, age; gender; and the items “standing up,” “washing oneself,” “swallowing,” “eating,” “defecation,” “understanding of routine,” “understanding of current season,” “abnormal muttering and laughing to oneself,” and “handling finances” were selected.

**Figure 2.**
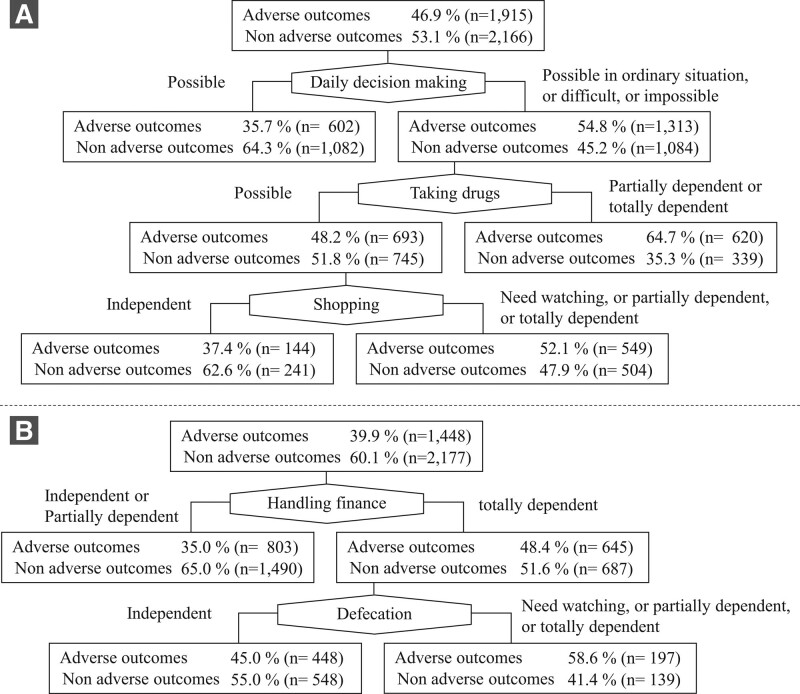
Decision tree models for prediction of adverse events within 1 year. (A) Model for those in support levels 1 and 2. (B) Model for those in care levels 1 and 2.

The decision tree model created using the variables selected by the stepwise logistic regression is presented in Figure 2. In support levels 1 and 2, a decision tree was created using the “daily decision making,” “taking drugs,” and “shopping’” items. Among those who scored both “daily decision making” item as other than “possible” and “taking drugs” item as other than “independent,” 64.7% had an adverse outcome. The accuracy of classification of the model was 61.1%. In care levels 1 and 2, a decision tree was created using the “handling finance,” and “defecation” items. Among those who scored the “shopping” item as “totally dependent” and the “defecation” item as other than “independent,” 58.6% had an adverse outcome. The accuracy of classification of the model was 61.7%.

## 4. Discussion

In this study, among the older adults who had received long-term care insurance certification with a mild disability level, to realize early intervention for high risk older adults, we tried to create simple prediction tools that predict the aggravation of the need for long-term care or death within 1 year. The accuracy of classification of the 2 decision trees created was about 60%, which is not practical accuracy. Therefore, the predictive tool of this study has a limited ability to predict the aggravation of the need for long-term care and the risk of death for all older adults. However, the decision tree of this study revealed that specific older adults group who fulfilled multiple criteria of the certification questionnaire faced an increased risk of the aggravation of the need for long-term care and death.

The variables selected in the decision tree were “daily decision making,” “taking drugs,” and “shopping” in support levels 1 and 2, and “handling finances” and “defecation” in care levels 1 and 2. In other words, all items except “defecation” were items of adaptation to social life. “Taking drugs,” “shopping,” and “handling finances” can be regarded as instrumental activities of daily living (IADLs). Because IADLs involve more complex tasks than ADLs, difficulties occur in IADLs before difficulties occur in ADLs among older adults.^[13,14]^ Therefore, it is considered that the IADLs items were selected as decision trees because of their potential to predict decline in ADLs, which is the main factor for aggravation of the level of care required. Regarding the order of difficulty of IADLs, it has been reported that managing health care, including taking drugs, is the greatest challenge and that managing finances is relatively easy.^[14]^ Considering this difficulty in IADLs, the results of decision trees that applied “taking drugs” in support levels 1 and 2, and “handling finances” in care levels 1 and 2 are reasonable. In addition, “daily decision making” selected in the decision tree for support levels 1 and 2 and “defecation” selected in the decision tree for care levels 1 and 2 are consistent with previous studies. ^[10,15]^ Sagari et al^[10]^ reported that “daily decision making” was related to the aggravation of the need for care after 2 years for those in support levels 1 and 2, similar to our study. Regarding “defecation,” Fusco et al^[15]^ reported that bowel incontinence in community dwelling frail elderly persons is a negative predictor of ADL ability.

Although the overall accuracy of the decision tree in this study is low, making it impractical to use it for all subjects, the novelty of this study is that we are able to identify a specific group more likely to experience adverse events within a year by identifying the overlapping effects of these risk factors. For example, in support levels 1 and 2, the overall rate of aggravation of the need for long-term care or death within 1 year was 46.9%; however, the risk increased to 64.7% (nearly 1.4 times) for those who scored both the “daily decision making” item as other than “possible” and the “taking drugs” item as other than “independent.” Similarly, in care levels 1 and 2, the risk of adverse events increased to nearly 1.5 times in cases where the “handling finance” item was “totally dependent” and “defecation” item was other than “independent.” Therefore, checking whether each of the 2 conditions is met at the support and care levels can be a very simple risk determination tool. However, almost 23.5% of people in support level (959/4081) and 9.3% in care level (336/3625) can use this tool to determine their risk of adverse events, and the risk of other people cannot be determined. Although the inability to apply this to all subjects is a major limitation, the findings of this study that the results of the 2 assessments can identify some older adults who are at a high risk of an aggravated need for long-term care or death within 1 year is very simple and useful.

For the identified high risk older adults, for example, when the municipality notifies the subject of the certification result, such measures should be taken as providing information to encourage the active use of long-term care insurance services such as outpatient and home-visit rehabilitation. According to a survey conducted by Omizu et al,^[16]^ 52% of those who were certified but did not use long-term care insurance services were in support level 1, and 37% were in support level 2. In addition, it has also been reported that approximately half of the nonusers mentioned above were independent in terms of ADLs but had difficulties with mobility and IADLs, and a certain number of people wanted to use long-term care insurance services but did not know how to use them.^[16]^ Encouraging the use of long-term care insurance services as early as possible for high risk older adults may lead to a reduction in the aggravation of the need for long-term care.

The results of this study suggest that variables such as “daily decision making,” “taking drugs,” “shopping,” “handling finances,” and “defecation” are associated with adverse events occurring within 1 year among older adults eligible for Japanese long-term care insurance. However, this study does not deny the relationship between other variables not selected in the decision tree (such as physical function and ADL functions other than defecation) and adverse events. To avoid overfitting and to generalize the results of this study, we used a decision tree with a limited number of variables to choose from. This means that “daily decision making,” “taking drugs,” “shopping,” “handling finances,” and “defecation” were comparatively advantageous variables for predicting adverse events.

The limitation of this study is that it does not deal with disease information and nursing care service usage that may be related to the aggravation of the level of nursing care needs. Adding these variables in the future may improve the accuracy of the prediction tool. Another limitation is that this study was a retrospective observational study, which makes it difficult to refer to causality. Therefore, it is unclear whether the predictive factors shown in this study directly affect the aggravation of the level of the need for long-term care and death. Further research is needed in the future.

## Acknowledgments

The authors are deeply grateful to Professor Seiji Yasumura of the Department of Public Health, Fukushima Medical University School of Medicine for his great support and cooperation in carrying out this research. We would also like to thank the people of Koriyama City Public Health Center for their cooperation in providing the data.

## Author contributions

**Conceptualization:** Takaaki Fujta, Kazuaki Iokawa.

**Data curation:** Takaaki Fujta.

**Formal analysis:** Takaaki Fujta.

**Funding acquisition:** Takaaki Fujta, Kazuaki Iokawa.

**Investigation:** Takaaki Fujta.

**Methodology:** Takaaki Fujta, Kazuaki Iokawa.

**Project administration:** Takaaki Fujta.

**Software:** Takaaki Fujta.

**Supervision:** Kazuaki Iokawa.

**Validation:** Takaaki Fujta.

**Visualization:** Takaaki Fujta.

**Writing – original draft:** Takaaki Fujta.

**Writing – review & editing:** Takaaki Fujta, Kazuaki Iokawa.
